# Supplementary material for: Best practices for improving alignment and variant calling on human sex chromosomes
Source: bioRxiv. 2025 Apr 30:2025.04.29.651297. Preprint. [Version 1] doi: 10.1101/2025.04.29.651297 (PMC12190741; doi:10.1101/2025.04.29.651297)
Supplement: Supplement 1 [file media-1.pdf]

Supplementary Materials

Supplementary Tables **2**

Table S1. Aligning reads to sex chromosome complement reference genome improves variant calling on the X chromosome compared to aligning to a default reference genome. 2

Table S2. Per sample performance metrics on X and Y. 4

Table S3. Genomic features on X and Y. 8

Table S2. Variants were introduced into simulated sequences from male individuals from 1000 Genomes. 9

Table S3. Effects of masking the X-transposed region (XTR). 13

Table S4. Effects of haploid calling. 15

Table S5. Counts of variants across regions of the X and Y. 16

Table S6. Using diploid-based filter thresholds for some filters on haploid chromosomes results in less accurate variant calling. 20

Supplementary Figures **22**

Supplementary Figure 1. Variant calling accuracy declines substantially in XTR when masking XTR on the Y chromosome for alignment. 22

Figure S2. The proportion of false positives and false negatives on chromosome X are similar to autosomal ranges while chromosome Y has an elevated proportion of false negatives. 23

Figure S3. Distribution of DP, AN, and QD are similar between autosomes and the X chromosome in females 24

Supplementary Tables

Table S1. Aligning reads to sex chromosome complement reference genome improves variant calling on the X chromosome compared to aligning to a default reference genome.

Average number of true positives, false positives, and false negatives on the X chromosome across 10 simulated female (XX) samples and 10 simulated male (XY) samples and across the Y chromosome in 10 simulated male (XY) samples using a default and sex chromosome complement alignment approaches. Chromosome X was split into PARs, XTR, and non-PARs without XTR and chromosome Y was split into XTR, and non-PARs without XTR.

Chr	Region	Joint Genotyping with	Average # TP		Average # FP		Average # FN	
			Default	SCC	Default	SCC	Default	SCC
X	PARs	10 Females (XX)	0	4909	0	2	5763	853
		10 Males (XY)	0	4980	0	3	5852	872
		10 Females (XX) + 10 Males (XY)	0	4939 (4910 avg F, 4969 avg M)	0	2 (2 avg F, 2 avg M)	5807 (5763 avg F, 5852 avg M)	867 (852 avg F, 882 avg M)
		20 Females (XX)	0	4947	0	2	5823	875
		20 Males (XY)	1	5105	0	2	6004	900
		20 Females (XX) + 20 Males (XY)	1 (0 avg F, 1 avg M)	5013 (4934 avg F, 5091 avg M)	0	2 (2 avg F, 2 avg M)	5914 (5823 avg F, 6004 avg M)	901 (888 avg F, 913 avg M)
	non PARs (minus XTR)	10 Females (XX)	97994	98007	127	127	5148	5135
		10 Males (XY)	66780	66780	17	16	4217	4217
		10 Females (XX) + 10 Males (XY)	82840 (98063 avg F, 67618 avg M)	82851 (98007 avg F, 67626 avg M)	63 (115 avg F, 11 avg M)	63 (115 avg F, 11 avg M)	4228 (5077 avg F, 3379 avg M)	4217 (5062 avg F, 3371 avg M)
		20 Females (XX)	97778	97789	114	114	5194	5183
		20 Males (XY)	66745	66746	17	17	4053	4051
		20 Females (XX) + 20 Males (XY)	82615 (97810 avg F, 67420 avg M)	82627 (97826 avg F, 67428avg M)	55 (99 avg F, 11 avg M)	55 (99 avg F, 11 avg M)	4267 (5159 avg F, 3376 avg M)	4256 (5143 avg F, 3368 avg M)
	XTR	10 Females (XX)	3767	4515	3	4	841	93
		10 Males (XY)	2171	2171	1	1	756	756
		10 Females (XX) + 10 Males (XY)	3043 (3744 F,	3586 (4513 F,	1 (2 F, 0 M)	2 (3 F, 0 M)	725 (864 F, 586 M)	181 (94 F, 268

			2342 M)	2659 M)				M)
		20 Females (XX)	3834	4563	3	3	818	89
		20 Males (XY)	2202	2202	0	0	761	761
		20 Females (XX) + 20 Males (XY)	3094 (3805 avg F, 2383 avg M)	3636 (4562 avg F, 2711 avg M)	1 (2 avg F, 0 avg M)	1 (2 avg F, 0 avg M)	713 (847 avg F, 560 avg M)	171 (90 avg F, 252 avg M)
Y	non PARs (minus XTR)	10 Males (XY)	2026	2026	2	2	857	857
		20 Males (XY)	2262	2262	2	2	902	902
	XTR	10 Males (XY)	103	103	1	1	36	36
		20 Males (XY)	138	138	1	1	51	51

Table S2. Per sample performance metrics on X and Y.

This is for 20 males and 20 females. 20 males were joint genotyped together and 20 females were joint genotyped together separately. Male X and Y non PARs called haploid. SCC aligned data.

Region	Sample size	Sample	# True Positives	# False Positives	# False Negatives	# Simulated
PARs	20 Males (XY)	NA06984	4639	1	915	5554
		NA06986	4921	0	815	5736
		NA06994	5123	1	823	5946
		NA07048	5205	6	947	6152
		NA07051	5016	4	986	6002
		NA07347	5031	1	941	5972
		NA07357	4722	1	795	5517
		NA10851	5042	6	850	5892
		NA11829	4983	2	954	5937
		NA11831	4959	1	854	5813
		NA11843	5297	1	850	6147
		NA11881	5214	3	812	6026
		NA11893	5293	6	982	6275
		NA11919	5253	0	929	6182
		NA11930	5115	0	831	5946
		NA11932	5138	2	882	6020
		NA11992	5468	1	953	6421
		NA11994	5594	3	930	6524
		NA12003	5130	2	985	6115
		NA12005	4950	2	975	5925
	20 Females (XX)	NA06985	5218	3	942	6161
		NA06989	4760	1	938	5699
		NA07000	5225	2	835	6061
		NA07037	5064	2	738	5803
		NA07056	5036	2	858	5895
		NA10847	4975	1	891	5867
		NA11830	4604	2	713	5318
		NA11832	4638	0	895	5533
		NA11840	5013	1	952	5966
		NA11892	4552	4	773	5326
		NA11894	5172	6	798	5971
		NA11918	5105	1	1072	6178
		NA11920	5015	2	921	5936
		NA11931	4693	2	911	5605
		NA11933	5003	1	715	5719
		NA11995	5170	3	934	6105
		NA12004	5114	2	859	5973
		NA12006	4666	1	902	5569
		NA12044	4890	1	897	5789

		NA12046	5028	5	965	5994
X non PARs (minus XTR)	20 Males (XY)	NA06984	67367	22	4087	71458
		NA06986	68227	20	4016	72246
		NA06994	66423	20	4237	70662
		NA07048	64626	14	3788	68419
		NA07051	66676	13	3963	70643
		NA07347	66338	20	4238	70579
		NA07357	68635	13	4350	72989
		NA10851	67037	10	3956	70997
		NA11829	65732	14	3972	69708
		NA11831	68217	16	4070	72291
		NA11843	68996	19	4294	73294
		NA11881	66046	13	4088	70138
		NA11893	67934	19	3993	71931
		NA11919	68243	16	4004	72251
		NA11930	64872	26	3811	68687
		NA11932	68634	14	3943	72579
		NA11992	64121	17	4110	68235
		NA11994	65956	17	4073	70033
		NA12003	64990	16	4113	69108
		NA12005	65858	16	3920	69781
	Females (XX)	NA06985	96616	116	4854	101473
		NA06989	98587	116	5144	103736
		NA07000	100040	104	5461	105505
		NA07037	98692	118	5354	104053
		NA07056	99161	118	5108	104274
		NA10847	97279	109	5077	102362
		NA11830	95996	124	4894	100895
		NA11832	98222	99	5392	103618
		NA11840	99263	125	4976	104244
		NA11892	96104	116	5166	101272
		NA11894	100663	100	5244	105913
		NA11918	96290	129	5152	101447
		NA11920	97972	123	5481	103458
		NA11931	96058	107	5484	101548
		NA11933	95590	103	5225	100820
		NA11995	98925	89	5210	104141
		NA12004	100001	117	5490	105495
		NA12006	96246	115	4760	101014
		NA12044	98269	123	5165	103437
		NA12046	95804	127	5028	100837
X XTR	20 Males (XY)	NA06984	2490	1	874	3364
		NA06986	2243	0	755	2998
		NA06994	1795	1	570	2365
		NA07048	2415	0	791	3206
		NA07051	2029	0	673	2702
		NA07347	2498	0	1026	3525

		NA07357	2441	1	900	3341
		NA10851	1653	0	371	2024
		NA11829	2483	2	937	3420
		NA11831	1760	1	568	2328
		NA11843	2370	0	911	3281
		NA11881	2217	0	736	2953
		NA11893	2192	0	860	3052
		NA11919	2272	0	671	2943
		NA11930	2503	1	1010	3514
		NA11932	2297	0	752	3049
		NA11992	2240	0	727	2968
		NA11994	1793	0	570	2363
		NA12003	2225	0	790	3016
		NA12005	2127	1	725	2852
	Females (XX)	NA06985	4419	2	61	4480
		NA06989	4573	4	93	4666
		NA07000	4752	6	100	4853
		NA07037	4859	3	97	4956
		NA07056	4535	2	93	4629
		NA10847	4789	2	112	4901
		NA11830	3982	4	82	4064
		NA11832	4512	1	84	4596
		NA11840	3775	2	73	3848
		NA11892	4992	3	91	5083
		NA11894	4707	4	97	4804
		NA11918	5157	7	115	5273
		NA11920	4549	3	84	4633
		NA11931	5371	5	93	5465
		NA11933	4307	3	77	4384
		NA11995	4375	2	76	4451
		NA12004	4779	2	103	4882
		NA12006	4364	1	70	4435
		NA12044	4230	2	82	4312
		NA12046	4236	5	90	4326
Y non PARs	20 Males (XY)	NA06984	1761	1	783	2544
		NA06986	1953	4	833	2787
		NA06994	2745	2	1053	3799
		NA07048	1812	3	795	2608
		NA07051	3144	4	1132	4277
		NA07347	1691	3	806	2498
		NA07357	1852	7	817	2669
		NA10851	1712	2	763	2475
		NA11829	3056	2	1099	4156
		NA11831	1656	0	751	2407
		NA11843	2396	2	905	3302
		NA11881	2961	2	1101	4062
		NA11893	2721	1	1075	3797

		NA11919	2999	5	1128	4128
		NA11930	2898	2	1034	3933
		NA11932	2970	3	1112	4082
		NA11992	2792	3	1105	3898
		NA11994	1852	1	801	2653
		NA12003	3136	3	1102	4239
		NA12005	1894	2	863	2758

Table S3. Genomic features on X and Y.

All analyses in this manuscript were performed in GRCh38. We lifted over XTR and amplicons using the UCSC liftover tool. PARs were obtained from Ensembl. X chromosome ampliconic region 3 failed liftover and ampliconic region 5 split into 4 regions.

Chromosome	Region	Coordinate (GRCh37/hg19)	Coordinate (GRCh38)
X	PAR1	60001 - 2699520	10001 - 2781479
	PAR2	154931044 - 155260560	155701383 - 156030895
	XTR	88193855 - 93193855	89140830 - 93428068
	Ampliconic region 1	48202745 - 48292983	48343310 - 48434604
	Ampliconic region 2	48976199 - 49062381	49157254 - 49162986
	Ampliconic region 3	51395467 - 51492862	N/A
	Ampliconic region 4	51775560 - 51966529	52032464 - 52223402
	Ampliconic region 5	52518132 - 53027386	52511000 - 52511492
			52489200 - 52501394
			52520509 - 52520537
			139870917 - 139871081
	Ampliconic region 6	55464117 - 55574172	55437684 - 55547739
	Ampliconic region 7	62335733 - 62495350	63185573 - 63193371
	Ampliconic region 8	70894117 - 71055682	71674267 - 71835832
	Ampliconic region 9	71941159 - 72325075	72721314 - 73105236
	Ampliconic region 10	100818723 - 100903977	101563740 - 101648990
	Ampliconic region 11	101435778 - 101774391	102180805 - 102519463
	Ampliconic region 12	103195105 - 103362341	103940531 - 104117650
Y	PAR1	10001 - 2649520	10001 - 2781479
	PAR2	59034050 - 59363566	56987321 - 57217415
	XTR1	2918085 - 6103152	3050044 - 6235111
	XTR2	6400947 - 6616754	6532906 - 6748713
	Ampliconic region 1	6103152 - 6400947	6235111 - 6532906
	Ampliconic region 2	7442522 - 10135224	7574481 - 10266944
	Ampliconic region 3	16096353 - 16170613	13984473 - 14058733
	Ampliconic region 4	17986973 - 18017094	15875093 - 15905214
	Ampliconic region 5	18271675 - 18537845	16159795 - 16425965
	Ampliconic region 6	19568146 - 21032220	17456266 - 18870334
	Ampliconic region 7	23467839 - 28561582	21305953 - 26415435

Table S2. Variants were introduced into simulated sequences from male individuals from 1000 Genomes.

Variants were introduced into simulated sequence reads using variant call data from 1000 Genomes males and females from individuals of European, African, and Asian descent.

Sample ID	Population	Super population	Sex
NA06984	CEU	EUR	Male
NA06986	CEU	EUR	Male
NA06994	CEU	EUR	Male
NA07048	CEU	EUR	Male
NA07051	CEU	EUR	Male
NA07347	CEU	EUR	Male
NA07357	CEU	EUR	Male
NA10851	CEU	EUR	Male
NA11829	CEU	EUR	Male
NA11831	CEU	EUR	Male
NA11843	CEU	EUR	Male
NA11881	CEU	EUR	Male
NA11893	CEU	EUR	Male
NA11919	CEU	EUR	Male
NA11930	CEU	EUR	Male
NA11932	CEU	EUR	Male
NA11992	CEU	EUR	Male
NA11994	CEU	EUR	Male
NA12003	CEU	EUR	Male
NA12005	CEU	EUR	Male
NA06985	CEU	EUR	Female
NA06989	CEU	EUR	Female
NA07000	CEU	EUR	Female
NA07037	CEU	EUR	Female
NA07056	CEU	EUR	Female
NA10847	CEU	EUR	Female
NA11830	CEU	EUR	Female
NA11832	CEU	EUR	Female
NA11840	CEU	EUR	Female
NA11892	CEU	EUR	Female
NA11894	CEU	EUR	Female
NA11918	CEU	EUR	Female

NA11920	CEU	EUR	Female
NA11931	CEU	EUR	Female
NA11933	CEU	EUR	Female
NA11995	CEU	EUR	Female
NA12004	CEU	EUR	Female
NA12006	CEU	EUR	Female
NA12044	CEU	EUR	Female
NA12046	CEU	EUR	Female
NA18486	YRI	AFR	Male
NA18498	YRI	AFR	Male
NA18501	YRI	AFR	Male
NA18504	YRI	AFR	Male
NA18507	YRI	AFR	Male
NA18510	YRI	AFR	Male
NA18516	YRI	AFR	Male
NA18519	YRI	AFR	Male
NA18522	YRI	AFR	Male
NA18853	YRI	AFR	Male
NA18856	YRI	AFR	Male
NA18865	YRI	AFR	Male
NA18868	YRI	AFR	Male
NA18871	YRI	AFR	Male
NA18874	YRI	AFR	Male
NA18877	YRI	AFR	Male
NA18879	YRI	AFR	Male
NA18908	YRI	AFR	Male
NA18910	YRI	AFR	Male
NA18915	YRI	AFR	Male
NA18488	YRI	AFR	Female
NA18489	YRI	AFR	Female
NA18499	YRI	AFR	Female
NA18502	YRI	AFR	Female
NA18505	YRI	AFR	Female
NA18508	YRI	AFR	Female
NA18511	YRI	AFR	Female
NA18517	YRI	AFR	Female
NA18520	YRI	AFR	Female
NA18523	YRI	AFR	Female

NA18858	YRI	AFR	Female
NA18861	YRI	AFR	Female
NA18864	YRI	AFR	Female
NA18867	YRI	AFR	Female
NA18870	YRI	AFR	Female
NA18873	YRI	AFR	Female
NA18876	YRI	AFR	Female
NA18878	YRI	AFR	Female
NA18881	YRI	AFR	Female
NA18907	YRI	AFR	Female
NA18530	CHB	EAS	Male
NA18534	CHB	EAS	Male
NA18536	CHB	EAS	Male
NA18543	CHB	EAS	Male
NA18544	CHB	EAS	Male
NA18546	CHB	EAS	Male
NA18548	CHB	EAS	Male
NA18549	CHB	EAS	Male
NA18557	CHB	EAS	Male
NA18558	CHB	EAS	Male
NA18559	CHB	EAS	Male
NA18561	CHB	EAS	Male
NA18562	CHB	EAS	Male
NA18563	CHB	EAS	Male
NA18572	CHB	EAS	Male
NA18603	CHB	EAS	Male
NA18605	CHB	EAS	Male
NA18606	CHB	EAS	Male
NA18608	CHB	EAS	Male
NA18609	CHB	EAS	Male
NA18525	CHB	EAS	Female
NA18526	CHB	EAS	Female
NA18528	CHB	EAS	Female
NA18531	CHB	EAS	Female
NA18532	CHB	EAS	Female
NA18533	CHB	EAS	Female
NA18535	CHB	EAS	Female
NA18537	CHB	EAS	Female

NA18538	CHB	EAS	Female
NA18539	CHB	EAS	Female
NA18541	CHB	EAS	Female
NA18542	CHB	EAS	Female
NA18545	CHB	EAS	Female
NA18547	CHB	EAS	Female
NA18550	CHB	EAS	Female
NA18552	CHB	EAS	Female
NA18553	CHB	EAS	Female
NA18555	CHB	EAS	Female
NA18560	CHB	EAS	Female
NA18564	CHB	EAS	Female

Table S3. Effects of masking the X-transposed region (XTR).

Masking XTR in the reference genome increases the number of false positives in XTR but does not impact variant calling on the rest of the X and Y chromosomes. Number of true positives, false positives, and false negatives in XTR, X non-PARs (excluding XTR), and Y non-PARs (excluding XTR) in males when aligning to a reference genome with just the PARs masked on the Y chromosome and PARs and XTR masked on the Y chromosome.

Region	Sample	# TPs		# FPs		# FNs	
		Y PARs masked	Y PARs + XTR masked	Y PARs masked	Y PARs + XTR masked	Y PARs masked	Y PARs + XTR masked
XTR	NA11831	1754	2228	1	33580	574	75
	NA11829	2468	3300	2	33180	952	88
	NA10851	1638	1934	0	33808	386	76
	NA07357	2422	3222	1	33113	919	85
	NA07347	2499	3366	0	33042	1026	128
	NA07051	2016	2554	0	33466	686	130
	NA07048	2395	3061	0	33302	811	119
	NA06994	1798	2271	1	33479	567	63
	NA06986	2241	2853	0	33335	757	113
	NA06984	2478	3220	1	33144	886	107
$X_{\text{non-PARs minus XTR}}$	NA11831	68072	68070	17	17	4216	4218
	NA11829	65576	65576	13	13	4130	4130
	NA10851	66915	66917	10	10	4080	4078
	NA07357	68508	68508	17	17	4479	4479
	NA07347	66168	66170	20	20	4410	4408
	NA07051	66517	66517	14	14	4124	4124
	NA07048	64505	64500	13	14	3910	3915
	NA06994	66257	66254	19	19	4403	4406
	NA06986	68067	68067	22	22	4177	4177
	NA06984	67216	67214	19	19	4240	4242
$Y_{\text{non-PARs minus XTR}}$	NA11831	1616	1616	0	1	750	750
	NA11829	2793	2793	0	1	1021	1021
	NA10851	1680	1680	1	2	752	752
	NA07357	1810	1810	6	7	815	815
	NA07347	1625	1625	3	4	803	803
	NA07051	2873	2873	5	6	1067	1067
	NA07048	1717	1717	2	4	777	777
	NA06994	2533	2533	2	5	984	984

	NA06986	1871	1871	1	2	829	829
	NA06984	1743	1743	2	3	770	770

Table S4. Effects of haploid calling.

Haploid calling on X and Y in males reduces false positives compared to diploid calling but does not affect false negatives. Performance metrics in X and Y non-PARs for 10 simulated male samples when variant calling in diploid and haploid modes.

Region	Sample	# TPs		# FPs		# FNs	
		Diploid	Haploid	Diploid	Haploid	Diploid	Haploid
$X_{non\text{-}PARs}$	NA11831	69836	69826	252	18	4780	4790
	NA11829	68060	68044	246	15	5066	5082
	NA10851	68564	68553	256	10	4454	4466
	NA07357	70950	70930	215	18	5377	5398
	NA07347	68676	68667	244	20	5427	5436
	NA07051	68543	68533	236	14	4799	4810
	NA07048	66908	66900	215	13	4712	4721
	NA06994	68066	68055	234	20	4959	4970
	NA06986	70323	70308	243	22	4919	4934
	NA06984	69706	69694	222	20	5114	5126
$Y_{non\text{-}PARs}$	NA11831	1649	1645	112	0	758	762
	NA11829	3065	3051	126	1	1090	1104
	NA10851	1712	1707	92	2	763	768
	NA07357	1847	1838	97	7	822	831
	NA07347	1684	1672	124	3	813	825
	NA07051	3133	3127	130	5	1143	1149
	NA07048	1816	1812	104	2	791	795
	NA06994	2739	2733	72	2	1059	1065
	NA06986	1946	1940	112	4	840	846
	NA06984	1765	1761	124	4	779	783

Table S5. Counts of variants across regions of the X and Y.

False negatives are elevated in regions of sequence similarity on the X but evenly dispersed on the Y chromosome**.** Performance metrics for the 10 simulated male and 10 simulated female samples by region.

**Region**	**Sex**	**Sample**	**# True Positives**	**# False Positives**	**# False Negatives**	**# Simulated**
PARs	Male	NA06984	4655	1	899	5554
		NA06986	4932	2	804	5736
		NA06994	5138	1	808	5946
		NA07048	5225	6	927	6152
		NA07051	5032	5	970	6002
		NA07347	5053	1	919	5972
		NA07357	4737	3	780	5517
		NA10851	5058	6	834	5892
		NA11829	4999	3	938	5937
		NA11831	4974	1	839	5813
	Females	NA06985	5215	3	945	6161
		NA06989	4759	2	939	5699
		NA07000	5232	2	828	6061
		NA07037	5068	2	735	5803
		NA07056	5026	3	868	5895
		NA10847	4980	2	886	5867
		NA11830	4604	1	713	5318
		NA11832	4632	0	901	5533
		NA11840	5021	4	944	5966
		NA11892	4554	5	771	5326
$X_{\text{non-PARs minus XTR and amplicons}}$	Males	NA06984	66791	19	4110	71531
		NA06986	67696	21	4068	69330
		NA06994	65843	19	4294	70496
		NA07048	63902	13	3789	72487
		NA07051	66170	14	4022	69801
		NA07347	65537	18	4263	70194
		NA07357	68124	17	4361	67695
		NA10851	66513	10	3981	70139
		NA11829	65302	13	4026	71766
		NA11831	67443	16	4085	70903
	Females	NA06985	95926	136	4631	100558
		NA06989	97785	133	5007	102793

		NA07000	99537	114	5249	104787
		NA07037	98161	141	5176	103339
		NA07056	98391	116	4930	103323
		NA10847	96775	124	4930	101707
		NA11830	95508	121	4722	100231
		NA11832	97730	112	5182	102913
		NA11840	98536	145	4779	103316
		NA11892	95575	120	4975	100550
X_{XTR}	Males	NA06984	2478	1	886	3364
		NA06986	2241	0	757	2998
		NA06994	1798	1	567	2365
		NA07048	2395	0	811	3206
		NA07051	2016	0	686	2702
		NA07347	2499	0	1026	3525
		NA07357	2422	1	919	3341
		NA10851	1638	0	386	2024
		NA11829	2468	2	952	3420
		NA11831	1754	1	574	2328
	Females	NA06985	4411	4	69	4480
		NA06989	4571	5	95	4666
		NA07000	4753	8	100	4853
		NA07037	4856	2	100	4956
		NA07056	4531	3	98	4629
		NA10847	4780	2	121	4901
		NA11830	3980	5	84	4064
		NA11832	4510	1	86	4596
		NA11840	3775	2	73	3848
		NA11892	4984	4	99	5083
$X_{Amplicons}$	Males	NA06984	425	0	130	555
		NA06986	371	1	109	480
		NA06994	414	0	109	523
		NA07048	603	0	121	724
		NA07051	347	0	102	449
		NA07347	631	2	147	778
		NA07357	384	0	118	502
		NA10851	402	0	99	501
		NA11829	274	0	104	378
		NA11831	629	1	131	760

	Females	NA06985	720	2	195	915
		NA06989	793	1	150	943
		NA07000	505	2	213	718
		NA07037	536	0	178	714
		NA07056	767	0	184	951
		NA10847	489	0	166	655
		NA11830	513	1	151	664
		NA11832	526	0	179	705
		NA11840	748	1	180	928
		NA11892	552	1	170	722
$Y_{\text{non-PARs minus XTR and amplicons}}$	Males	NA06984	1607	2	714	2321
		NA06986	1727	1	763	2491
		NA06994	2214	2	855	3070
		NA07048	1559	2	708	2268
		NA07051	2521	5	928	3450
		NA07347	1508	3	742	2251
		NA07357	1666	6	744	2410
		NA10851	1564	1	686	2250
		NA11829	2459	0	891	3351
		NA11831	1462	0	684	2146
Y_{XTR}	Males	NA06984	18	2	13	2513
		NA06986	69	3	17	2701
		NA06994	200	0	81	3518
		NA07048	95	0	18	2495
		NA07051	254	0	82	3941
		NA07347	47	0	22	2429
		NA07357	28	1	16	2625
		NA10851	27	1	16	2432
		NA11829	258	1	83	3815
		NA11831	29	0	12	2366
Y_{ampicons}	Males	NA06984	136	0	56	192
		NA06986	144	0	66	210
		NA06994	319	0	129	448
		NA07048	158	0	69	227
		NA07051	352	0	139	491
		NA07347	117	0	61	178
		NA07357	144	0	71	215
		NA10851	116	0	66	182

		NA11829	334	0	130	464
		NA11831	154	0	66	220

Table S6. Using diploid-based filter thresholds for some filters on haploid chromosomes results in less accurate variant calling.

Average number of true positives, false positives, and false negatives on the sex chromosomes for 10 simulated males and 10 simulated females using diploid- and haploid-based thresholds for total depth (DP), allele number (AN), and QualByDepth (QD).

Filter	Sex	Chromosome	Threshold	Average # TP	Average # FP	Average # FN
DP	Males	$X_{non\text{-}PARs}$	Diploid based (67 and 201)	16082	2	57844
			Haploid based (23 and 69)	58662	24	15263
		$Y_{non\text{-}PARs}$	Diploid based (67 and 201)	685	1	2337
			Haploid based (23 and 69)	1944	7	1103
	Females	$X_{non\text{-}PARs}$	Diploid based (67 and 201)	103705	210	4045
			Haploid based (23 and 69)	771	9	106689
AN	Males	$X_{non\text{-}PARs}$	1	71695	27	2230
			2	71692	27	2233
			3	71687	27	2237
			4	71676	27	2248
			5	71655	27	2270
			10	67042	23	6883
			15	0	0	73927
			20	0	0	73927
		$Y_{non\text{-}PARs}$	1	2588	10	433
			2	2586	10	434
			3	2583	10	437
			4	2577	10	443
			5	2569	10	452
			10	2333	8	688
			15	0	0	3022
			20	0	0	3022
	Females	$X_{non\text{-}PARs}$	1	104803	222	2946
			2	104803	222	2946
			3	104802	222	2948
			4	104802	222	2948
			5	104800	222	2950

			10	104788	221	2962
			15	104702	220	3048
			20	104259	217	3491
QD	Males	$X_{non\text{-}PARs}$	1	71695	27	2230
			1.5	71695	27	2230
			2	71695	27	2230
			12	71691	21	2234
			16	71684	19	2241
			20	71637	17	2287
			28	54357	11	19568
		$Y_{non\text{-}PARs}$	1	2588	10	433
			1.5	2588	10	433
			2	2586	10	433
			12	2586	4	435
			16	2584	3	436
			20	2578	2	442
			28	1971	1	1049
	Females	$X_{non\text{-}PARs}$	1	104803	219	2946
			1.5	104803	212	2946
			2	104801	192	2949
			12	100586	13	7164
			16	90508	8	17242
			20	74247	3	33503
			28	34839	1	72911

Table S7. Sex chromosome complement specific variants in genes with clinical relevance.

For each gene in which we identified novel variants, we looked in the GWAS Catalog, OMIM phenotypes, and ClinVar to identify whether the gene had previously been implicated in human disease.

**Gene**	**Var**	**GWAS Catalog Risk Variant Reported Trait (gene)**	**OMIM Phenotypes (gene)**	**ClinVar (gene)**
PLCXD1	139	-	-	96 Pathogenic Variants; Conditions: Autism, Schizophrenia, Intellectual Disability
GTPBP6	114	-	-	96 Pathogenic Variants; Conditions: Autism, Schizophrenia, Intellectual Disability
PPP2R3B	1	-	-	97 Pathogenic Variants; Conditions: Autism, Schizophrenia, Intellectual Disability
SHOX	195	Severe COVID-19 infection, Vaginal microbiome	Langer mesomelic dysplasia, Leri-Weill dyschondrosteosis, Short stature, idiopathic familial	147 Pathogenic Variants; Conditions: Autism, Schizophrenia, Intellectual Disability, SHOX-related short stature, Leri-Weill dyschondrosteosis, Langer mesomelic dysplasia syndrome
RPL14P5	4	Schizophrenia, vaginal microbiome	-	-
CRLF2	185	Severe COVID-19 infection, Schizophrenia, Eosinophil counts, Eosinophil percentage of white cells	-	100 Pathogenic Variants; Conditions: Autism, Schizophrenia, Intellectual Disability

CSF2RA	320	Mitochondrial heteroplasmy, Granulocyte-macrophage-colony stimulating factor receptor subunit alpha levels, Eosinophil counts, Interleukin-3 receptor subunit alpha levels, Basophil count, Basophil percentage of leukocytes	Surfactant metabolism dysfunction, pulmonary, 4	115 Pathogenic Variants; Conditions: Autism, Schizophrenia, Intellectual Disability, Surfactant metabolism dysfunction, pulmonary, 4, CSF2RA-related condition
RNA5SP498	1	-	-	-
IL3RA	219	Interleukin-3 receptor subunit alpha levels, Basophil count, Basophil percentage of leukocytes	-	96 Pathogenic Variants; Conditions: Autism, Schizophrenia, Intellectual Disability
SLC25A6	30	-	-	95 Pathogenic Variants; Conditions: Autism, Schizophrenia, Intellectual Disability
LINC00106	36	-	-	95 Pathogenic Variants; Conditions: Autism, Schizophrenia, Intellectual Disability
ASMTL-AS1	46	-	-	95 Pathogenic Variants; Conditions: Autism, Schizophrenia, Intellectual Disability
ASMTL	329	N-acetylserotonin O-methyltransferase-like protein levels	-	95 Pathogenic Variants; Conditions: Autism, Schizophrenia, Intellectual Disability
P2RY8	439	Severe COVID-19 infection, Lymphocyte percentage of leukocytes, Eosinophil counts, Eosinophil percentage of leukocytes, Lymphocyte count, Erythrocyte volume, Mean reticulocyte volume, Erthrocyte volume	-	95 Pathogenic Variants; Conditions: Autism, Schizophrenia, Intellectual Disability

AKAP17A	37	Lymphocyte count	-	96 Pathogenic Variants; Conditions: Autism, Schizophrenia, Intellectual Disability
ASMT	144	-	-	96 Pathogenic Variants; Conditions: Autism, Schizophrenia, Intellectual Disability
DHRSX	1307	Severe COVID-19 infection, Brain language network functional connectivity	Congenital disorder of glycosylation, type 1DD	101 Pathogenic Variants; Conditions: Autism, Schizophrenia, Intellectual Disability, Congenital disorder of glycosylation, type 1DD
DHRSX-IT1	8	-	-	-
ZBED1	58	-	-	99 Pathogenic Variants; Conditions: Autism, Schizophrenia, Intellectual Disability
CD99P1	237	Adolescent idiopathic scoliosis	-	-
LINC00102	6	-	-	98 Pathogenic Variants; Conditions: Autism, Schizophrenia, Intellectual Disability
CD99	215	Docking protein 1 levels, erythrocyte volume	-	98 Pathogenic Variants; Conditions: Autism, Schizophrenia, Intellectual Disability
XG	154	Glycoprotein Xg levels, Erythrocyte volume	XG blood group system, Xg(a-) phenotype	208 Pathogenic Variants; Conditions: Autism, Schizophrenia, Kleinfelter's Syndrome, Intellectual disability, Turner's Syndrome, Trisomy X syndrome, 46,XX sex reversal 1, Hypotonia, Neurodevelopmental disorder
MXRA5	4	Hip circumference, Severe COVID-19 infection, Intraocular pressure, Pulse pressure	-	209 Pathogenic Variants; Conditions: Autism, Schizophrenia, Klinefelter syndrome, Turner syndrome, Intellectual Disability, Intellectual Developmental Disorder, Inborn genetic diseases,
PRKX	2	Thyroid stimulating hormone levels, Blood urea nitrogen levels	-	204 Pathogenic Variants; Conditions: Autism, Schizophrenia, Klinefelter syndrome, Turner syndrome, Intellectual Disability, Neurodevelopmental disorder, Trisomy X syndrome, 46,XX sex reversal 1, Hypotonia

NLGN4X	2	Schizophrenia, Neuroticism, RBC levels of ADP, RBC levels of citrate, Neuroligin-4 X-linked levels, Insomnia, memory decline, circulating fibrinogen levels, Alzheimer's disease, tuberculosis	Autism susceptibility, X-linked 2, Intellectual developmental disorder, X-linked	204 Pathogenic Variants; Conditions: Autism, Schizophrenia, Klinefelter syndrome, Turner syndrome, Intellectual Disability, Neurodevelopmental disorder, Trisomy X syndrome, 46,XX sex reversal 1, Hypotonia
ANOS1	18	Severe COVID-19 infection, Tumor protein p53-inducible protein 13 levels, Serum levels of protein TP53I13, Nipple retraction (>= grade 2) in breast cancer treated with radiotherapy, Facial morphology (factor 17, height of vermillion upper lip)	Hypogonadotropic hypogonadism 1 with or without anosmia (Kallmann syndrome 1)	250 Pathogenic Variants; Conditions: Autism, Schizophrenia, Intellectual Disability, Hypogonadotropic hypogonadism 1 with or without anosmia, Klinefelter syndrome, Inborn genetic diseases, Turner syndrome, Trisomy X syndrome, 46,XX sex reversal 1, Hypotonia, Neurodevelopmental disorder
CTPS2	2	Suicide, Neuroticism	-	160 Pathogenic Variants; Conditions: Scizophrenia, Autism, Klinefelter syndrome, Turner syndrome, Trisomy X syndrome, 46,XX sex reversal 1, Hypotonia, Neurodevelopmental disorder, Syndromic X-linked intellectual disability Lubs type

DMD	1	COVID-19, vaginal microbiome, Body mass index, breast carcinoma, tuberculosis, waist circumference, influenza A, anxiety, gut microbiome, temporomandibular joint disorder, major depressive disorder, educational attainment, blood urea nitrogen, ovarian carcinoma, metabolite levels, migraine, bipolar disorder and schizophrenia, weight, facial morphology, electroencephalogram vigilance, memory performance, itch intensity from mosquito bite, half-life of apixaban, Alzheimer's disease, Type 2 diabetes, systolic blood pressure, sphingosine 1-phosphate RBC levels, mortality, ankle-brachial index, octadecenoic acid RBC levels, ovarian carcinoma, Severe COVID-19 infection, serum levels of protein TENM3, periodontitis	Becker muscular dystrophy, Cardiomyopathy, dilated, 3B, Duchenne muscular dystrophy	2840 Pathogenic Variants; Conditions: Klinefelter syndrome, Duchenne muscular dystrophy, Becker muscular dystrophy, Polymicrogyria, Congenital adrenal hypoplasia, X-linked, Qualitative or quantitative defects of dystrophin, Abnormality of the musculature, Muscular dystrophy, Myopathy, Cardiovascular phenotype, McLeod neuroacanthocytosis syndrome, Elevated circulating creatine kinase concentration, Dilated cardiomyopathy 3B, Colorectal cancer, DMD-related disorder, Highly elevated creatine kinase, Intermediate muscular dystrophy, Turner syndrome, Ornithine carbamoyltransferase deficiency, Neurodevelopmental disorder, Syndromic X-linked intellectual disability Lubs type, Primary familial hypertrophic cardiomyopathy, Fanconi anemia complementation group A, Becker muscular dystrophy, atypical

KLHL4	1	Severe COVID-19 infection, Bone mineral density, Adolescent idiopathic scoliosis, Alzheimer's disease, Metabolite levels, Alcoholic pancreatitis, memory decline	-	145 Pathogenic Variants; Conditions: Autism, Schizophrenia, Klinefelter syndrome, Turner syndrome, Trisomy X syndrome, 46,XX sex reversal 1, Hypotonia, Syndromic X-linked intellectual disability Lubs type, Heterotaxy, visceral, 1, X-linked
PCDH11X	349	Alzheimer's disease, Severe COVID-19 infection, Metabolite levels, Tuberculosis	-	143 Pathogenic Variants; Conditions: Autism, Schizophrenia, Klinefelter syndrome, Turner syndrome, Intellectual Disability, Neurodevelopmental disorder, Trisomy X syndrome, 46,XX sex reversal 1, Hypotonia, Xq21.32q23 deletion, Developmental and epileptic encephalopathy, 9, Syndromic X-linked intellectual disability Lubs type, Heterotaxy, visceral, 1, X-linked
HTR2C	5	Educational attainment	-	154 Pathogenic Variants; Conditions: Autism, Schizophrenia, Klinefelter syndrome, Turner syndrome, Trisomy X syndrome, 46,XX sex reversal 1, Hypotonia, Syndromic X-linked intellectual disability Lubs type, Bone mineral density quantitative trait locus 18, Heterotaxy, visceral, 1, X-linked, Premature ovarian failure
SPRY3	36	Body mass index, Triglycerides, Idiopathic osteonecrosis of the femoral head, Total cholestorol levels, Low density liproprotein cholestorol levels, Hemoglobin, Mean corpuscular volume, Mean corpuscular homoglobin, Hematocrit, Red blood cell count, Red cell distribution width, Pyruvate levels in blood donors	-	104 Pathogenic Variants; Conditions: Autism, Schizophrenia, Kleinfelter's Syndrome, Ectodermal dysplasia and immunodeficiency 1, Immunodeficiency 33, Immunodeficiency 47, Incontinentia pigmenti syndrome, Decreased circulating antibody concentration, Splenomegaly

AMD1P2	2	Pyruvate levels in blood donors	-	-
VAMP7	126	Eosinophil counts, Eosinophil percentage of leukocytes	-	93 Pathogenic Variants; Conditions: Autism, Schizophrenia, Kleinfelter's Syndrome, Ectodermal dysplasia and immunodeficiency 1, Immunodeficiency 33, Immunodeficiency 47, Incontinentia pigmenti syndrome, Decreased circulating antibody concentration, Splenomegaly
ELOCP24	1	Eosinophil counts, Eosinophil percentage of leukocytes	-	-
TRPC6P	10	-	-	-
IL9R	26	Eosinophil counts, Eosinophil percentage of leukocytes	-	81 Pathogenic Variants; Conditions: Autism, Schizophrenia, Klinefelter syndrome, Immunodeficiency 47, Immunodeficiency 33, Ectodermal dysplasia and immunodeficiency 1, Incontinentia pigmenti syndrome, Decreased circulating antibody concentration, Splenomegaly, Autoimmune thrombocytopenia
AL158055.1	1	-	-	-
AC073614.1	1	-	-	-
AL121872.1	22	-	-	-
AL683807.1	165	-	-	-
AL672277.1	18	-	-	-
AL954722.1	16	-	-	-
AL683807.2	12	-	-	-

Supplementary Figures

Figure S1. Variant calling accuracy declines substantially in XTR when masking XTR on the Y chromosome for alignment.

Box plots of the number of called variants over the number of simulated variants in XTR across 10 simulated male (XY) individuals using different alignment approaches. We aligned simulated reads from 10 male (XY) samples to two different versions of the reference genome - one where the PARs on the Y chromosome are hard masked (SCC), and another with both PARs and XTR hard masked on the Y chromosome (SCC + Y-XTR masked) - and called variants and hard filtered using GATKs best practices protocol. We called variants in XTR for the SCC aligned data as haploid for both the X and Y chromosomes and for the SCC + Y-XTR masked aligned data we called variants in XTR as diploid on chromosome X. We then counted the number of variants called in XTR and compared that to the number of simulated variants in XTR for X and Y for each simulated sample. Since variants were called only on X in the approach with XTR hard masked on Y, we summed the number of X and Y chromosome simulated variants.

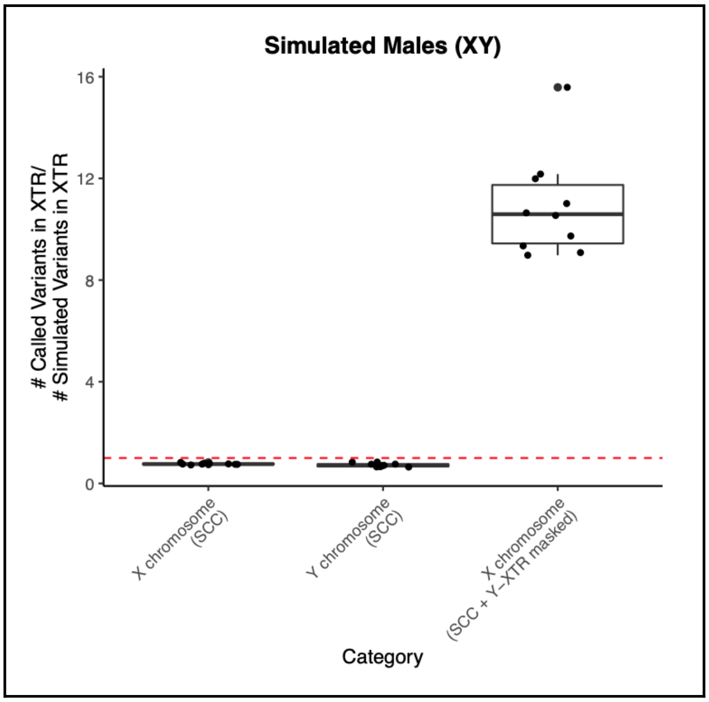

Figure S2. Proportion of false positives and false negatives on chromosome X and chromosome Y.

The proportion of false positives and false negatives on chromosome X are similar to autosomal ranges while chromosome Y has an elevated proportion of false negatives. Box plots of the proportion of false positives to the total number of simulated variants across A) 10 simulated female (XX) individuals and B) 10 simulated male (XY) individuals, and the proportion of false negatives to the total number of simulated variants across C) 10 simulated female (XX) individuals and D) 10 simulated male (XY) individuals across the autosomes and sex chromosomes. All data was aligned to a reference genome informed on the sex chromosome complement of the sample, diploid calling mode was performed on the autosomes and the X chromosome in simulated females, and haploid calling mode was performed on the X and Y chromosomes in simulated males. GATK hard filters with recommended thresholds were implemented for all chromosomes. Joint genotyping was performed across 10 simulated females and 10 simulated males separately. Highest and lowest proportions of false positives and false negatives are annotated with dashed horizontal red lines.

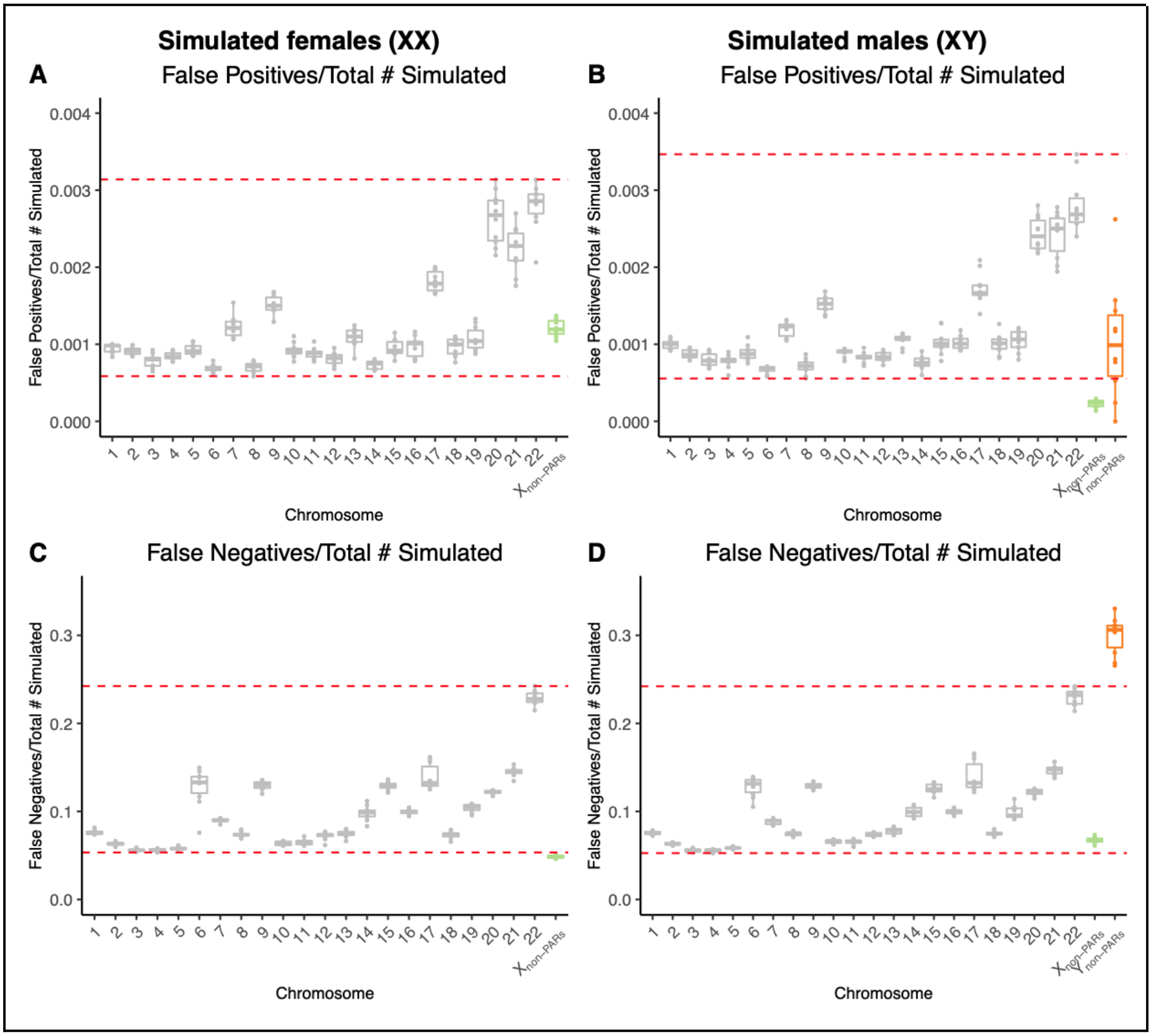

Figure S3. Distribution of DP, AN, and QD are similar between autosomes and the X chromosome in females

Distributions of variants (top) and the number of true positives and false positives for different filtering thresholds for autosomes, X non-PARs, and Y non-PARs across simulated female (XX) samples for A) total depth, B) allele number, and C) QualByDepth. Chromosome 8 was used to represent the autosomes. Vertical red dashed lines represent filter thresholds based on the autosomes.

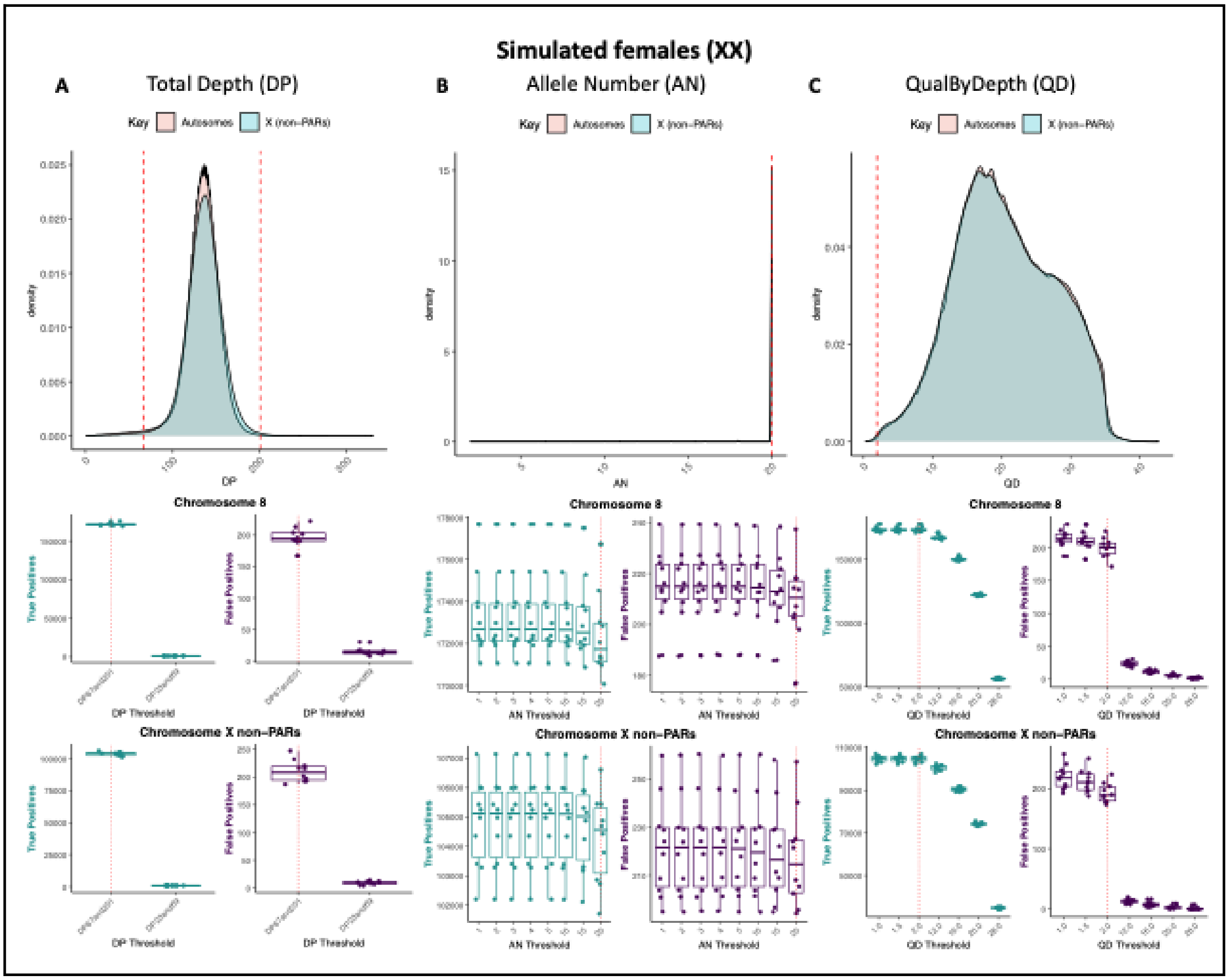

Figure S4. Comparing 10 vs 20 sample results.

The proportion of false positives to the total number of simulated variants and false negative relative to the total number of simulated variants are similar when joint genotyping across 10 and 20 samples. Box plots of the proportion of false positives to the total number of simulated variants across A) simulated females (XX) and B) simulated males (XY), and the proportion of false negatives to the total number of simulated variants across C) simulated females (XX), D) simulated males (XY) when joint genotyping 10 and 20 samples.

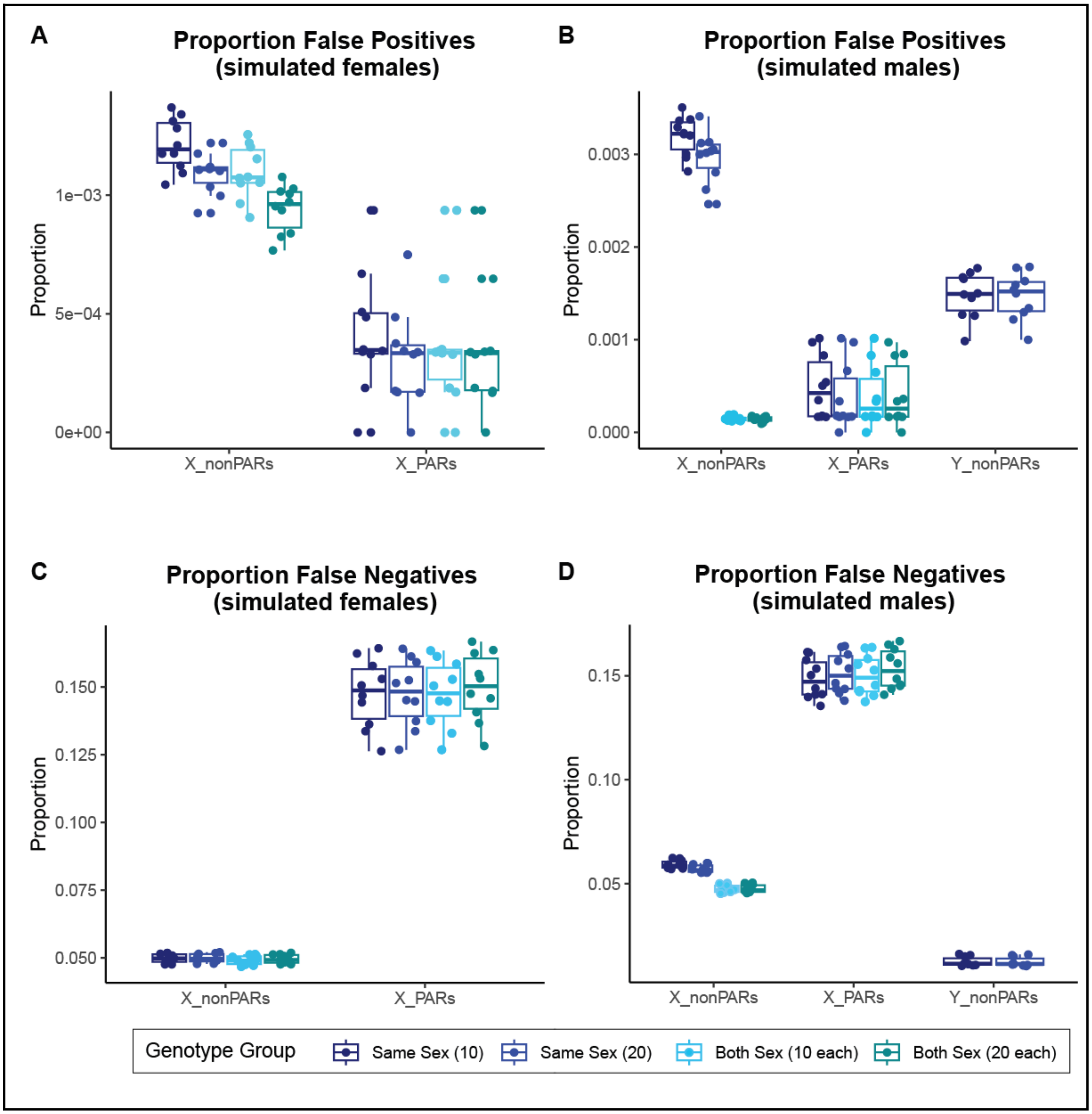

Figure S5. Proportions of false positives and false negatives are similar across simulated individuals from different ancestries.

Overall, we observed no large differences in the proportion of false positives and false negatives across simulated individuals from different ancestries. In females the proportion of false positives on the PARs are the highest for simulated African samples (average proportion of false positives = 0.0007) but slightly lower for the simulated Asian (average proportion of false positives = 0.0005) and European samples (average proportion of false positives = 0.0004; **Figure S5A**). A similar pattern was observed in males, where the average proportion of false positives is 0.0006 across simulated African and Asians, and 0.0004 across simulated Europeans samples (**Figure S5B**). The proportion of false positives on X non-PARs in females is equal across simulated Asian and European samples (average proportion of false positives = 0.0011) but slightly lower across the simulated African samples (average proportion of false positives = 0.0008; **Figure S5A**). For males, the average proportion of false positives on X non-PARs is equal across all simulated ancestries (average proportion of false positives = 0.0002). On the Y non-PARs, the proportion of false positives is highest for simulated African samples (average proportion of false positives = 0.0011) but slightly lower and equal for the simulated Asian and European samples (average proportion of false positives = 0.0008; **Figure S5B**).

Similar to false positives, the proportion of false negatives on the PARs are highest for simulated African samples (average proportion of false positives = 0.1661) but slightly lower for the simulated Asian (average proportion of false positives = 0.1627) and European female samples (average proportion of false positives = 0.1502; **Figure S5C**). This pattern was also observed across males on the PARs (average proportion of false positives Africans = 0.1644; average proportion of false positives Asians = 0.1622; average proportion of false positives Europeans = 0.1500; **Figure S5D**). On X non-PARs, the proportion of false negatives is lowest across simulated African samples and slightly higher for both Asian and European samples for both simulated females (average proportion of false positives in simulated Africans = 0.0445; average proportion of false positives in simulated Asians = 0.0513; average proportion of false positives in simulated Europeans = 0.0490) and males (average proportion of false positives in simulated Africans = 0.0613; average proportion of false positives in simulated Asians = 0.0692; average proportion of false positives in simulated Europeans = 0.0652; **Figure S5D**). A similar pattern was observed on the Y non-PARs (average proportion of false positives in simulated Africans = 0.2678; average proportion of false positives in simulated Asians = 0.2704; average proportion of false positives in simulated Europeans = 0.2881; **Figure S5D**).

Box plots of the proportion of false positives to the total number of simulated variants across A) 20 simulated females (XX) and B) 20 simulated males (XY), and the proportion of false negatives to the total number of simulated variants across C) 20 simulated females (XX) and D) 20 simulated males (XY) for simulated African, Asian, and European samples.

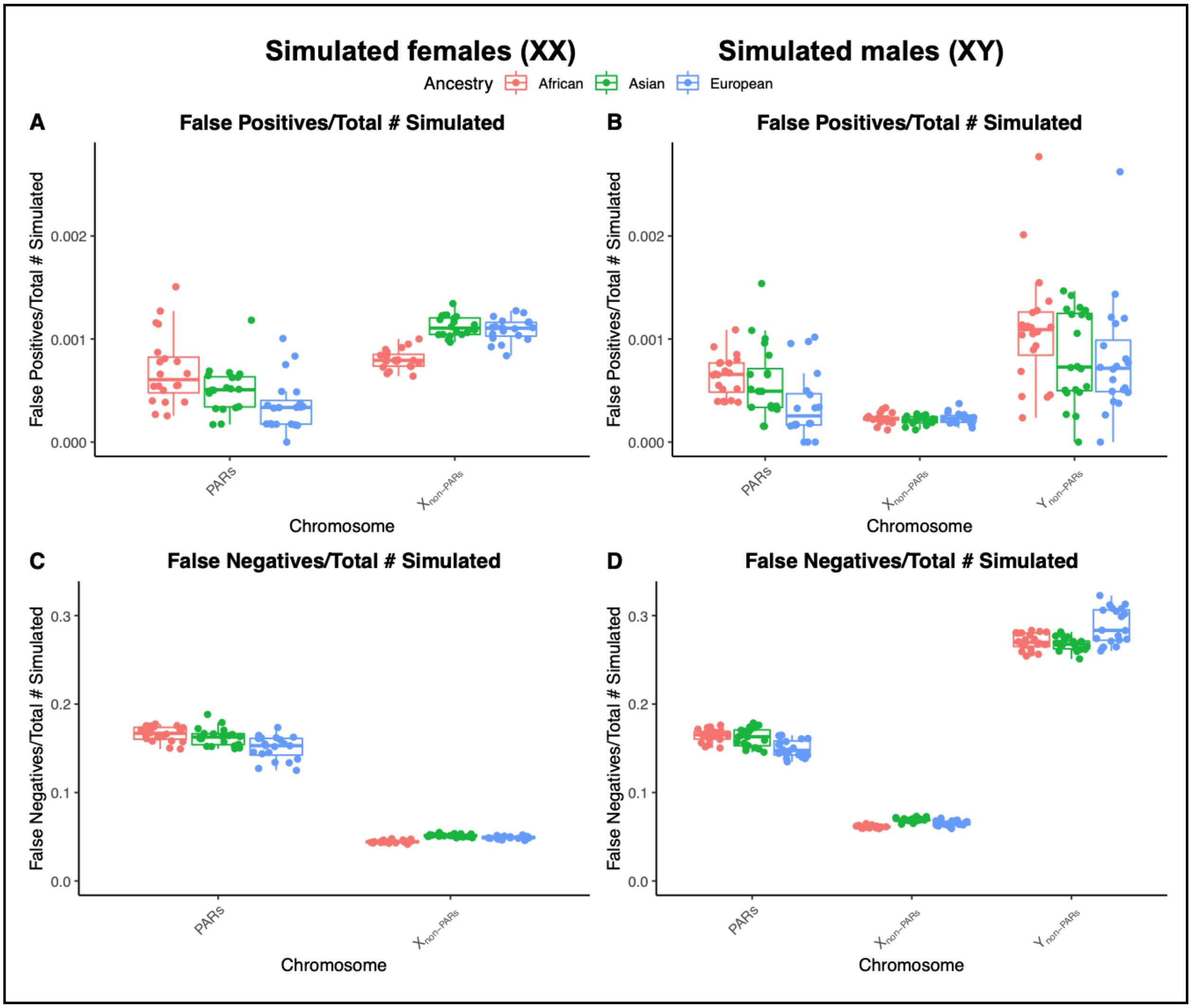

Simulated females (XX)
Simulated males (XY)
Ancestry
African
Asian
European
A
False Positives/Total # Simulated
B
False Positives/Total # Simulated
C
False Negatives/Total # Simulated
D
False Negatives/Total # Simulated
False Positives/Total # Simulated
False Negatives/Total # Simulated
PARs
X non-PARs
Y non-PARs
Chromosome
